# Macular edema after surgery to treat rhegmatogenous retinal detachment: 1-year follow-up, incidence, and associated risk factors

**DOI:** 10.5935/0004-2749.2022-0335

**Published:** 2024-03-27

**Authors:** Caroline Thais Machry Finger, Gabriela Maliska, Sérgio Brillinger Novello

**Affiliations:** 1 Department of Ophthalmology, Hospital Regional de São José, São José, SC, Brazil

**Keywords:** Macular edema, Retinal detachment, Vitrectomy, Tomography, optical coherence, Incidence, Risk factors

## Abstract

**Purpose:**

To clarify the postoperative incidence of macular edema in patients undergoing surgery to repair rhegmatogenous retinal detachment and identify the associated risk factors.

**Methods:**

In this prospective, observational study, 79 patients who underwent surgery to correct rhegmatogenous retinal detachment using pars plana vitrectomy with silicone oil injection were analyzed. Patients were followed up postoperatively at 7, 30, 90, 180, and 365 days. At each visit, optical coherence tomography was performed to assess the presence or absence of macular edema. were analyzed as possible risk factors for macular edema: age, sex, macular status (attached or detached), presence of vitreoretinal proliferation, history of previous intraocular surgery, reported time of symptoms suggestive of rhegmatogenous retinal detachment up to the date of surgery, and the surgical modality performed.

**Results:**

The 1-year macular edema prevalence rate was 26.6%. In the adjusted analysis, older patients had a higher risk of macular edema, and each 1-year increase in age increased the risk of macular edema by 6% (95% confidence interval = 1.00-1.12). The macular status, vitreoretinal proliferation, the surgical technique used, prior intraocular surgery, and the intraocular lens status were not identified as risk factors. However, the incidence of macular edema increased up to 180 days after surgery, peaking at 10.6%, and then decreased until 365 days after surgery.

**Conclusion:**

Macular edema was a common complication after surgery to treat rhegmatogenous retinal detachment, with its incidence peaking between 30 and 180 days after surgery. Age was an important risk factor for macular edema in this cohort.

## INTRODUCTION

Rhegmatogenous retinal detachment (RRD) occurs when fluid from the vitreous cavity infiltrates the subretinal region through a tear in the retina, resulting in anatomical separation between the neurosensory layer and retinal pigment epithelium^(1^,^2)^. RRD is an important cause of low visual acuity, and it carries a risk of irreversible vision loss. Its reported incidence in the literature is 13.3 cases per 100,000 inhabitants^(3^-^5)^.

The treatment of RRD consists of reattaching the retina and sealing the retinal tear^(6^,^7)^. However, even after surgery with an adequate technique and good anatomical results, visual acuity might only recover partially or even decline in the late postoperative period. Macular edema (ME) is one possible cause of low visual acuity, but it is treatable after diagnosis^(8^,^9)^. Its pathophysiology is not fully understood, but the most plausible theory states that ME results from a low-intensity, subclinical inflammatory process in which pro-inflammatory substances (e.g., cytokines and prostaglandins) induce loss of the blood-aqueous barrier and the consequent accumulation of intraretinal fluid in the macular region^(10^,^11)^.

With the advent of optical coherence tomography (OCT), an in vivo and non-invasive assessment of the retinal layers is possible. OCT is an extremely effective tool for detecting postoperative structural changes in the macular region, such as the presence of intraretinal fluid, which can be difficult to detect by biomicroscopy or fluorescein angiography^(8^,^12)^.

This study investigated the postoperative incidence of ME in patients undergoing surgery to repair RRD using OCT and assessed the potential risk factors.

## METHODS

This prospective, observational study was approved by the ethics committee of *Instituto de Cardiologia de Santa Catarina* and conducted in accordance with the principles of the Declaration of Helsinki.

In total, 110 patients with RRD undergoing surgical treatment in the Ophthalmology Department of Hospital Regional de São José (HRSJ, Santa Catarina, Brazil) during in 2021 or 2022 were eligible for enrollment. Patients with recurrent retinal detachment, a history of macular disease, uveitis, ocular trauma, endophthalmitis, corneal or lens alterations that prevented the performance of OCT, a history of retinopexy (because of the low number of eyes in the final sample), and no interest in participating in the research study were excluded from the analysis. Finally, 79 patients met the inclusion criteria for sample composition.

The patients were evaluated by OCT using the Spectralis^®^ device (Heidelberg Engineering GmbH, Heidelberg, Germany) at predetermined postoperative intervals: 7, 30, 90, 180, and 365 days. At each visit, OCT was performed to assess the presence of ME. The accumulation of fluid between the retinal layers in the macular region was established as a diagnostic criterion for ME. This study did not only consider cases of cystoid ME.

The following variables of interest were assessed: age, sex, macular status immediately before surgical correction (attached or detached), presence of proliferative vitreoretinopathy (PVR), the reported duration between the appearance of symptoms suggestive of RRD and surgery, the surgical procedure [pars plana vitrectomy (PPV) with silicone oil (SO) injection combined with phacoemulsification plus an intraocular lens implant or PPV with SO injection], history of previous eye surgery, and the intraocular lens status after RRD correction (phakic, aphakic, or pseudophakic).

All patients underwent surgical correction of RRD at HRSJ. Surgery was performed by different retina surgeons, but the same vitrectomy system (Eva Dorc^®^, Zuidland, the Netherlands) was used in all retinal surgeries. No restriction regarding the surgical technique used to perform vitrectomy (e.g., use of perfluorocarbon, type of buffering agent, use of retinotomy) was implemented.

Data were tabulated and stored in the Excel^®^ program (Microsoft, Redmond, WA, USA) and analyzed descriptively and inferentially using IBM SPSS version 20.0 (IBM, Armonk, NY, USA). All variables were analyzed descriptively using the mean and standard deviation and/or absolute and relative frequencies. Sociodemographic data and other variables of interest were analyzed in the total sample and used to characterize the patients.

In the sample characterization, the Mann-Whitney U test was used to compare age and the incidence of ME over time. To identify differences in sex, PVR, previous surgery, and the incidence of ME between the groups, the chi-squared test was used. Fisher’s exact test was used to investigate differences in the macular status, duration of symptoms, type of surgery performed, intraocular lens status (phakic/pseudophakic/phakic), and incidence of ME between the groups.

To identify possible risk and protective factors for the incidence of ME, binary logistic regression was used. Two models were analyzed, including unadjusted and adjusted models. For the adjusted model, the backward selection method, which eliminates variables from the model that may not explain variations in the dependent variable, was used. Thus, the model with the best fit, as verified using the Hosmer-Lemeshow test, served as the final model of the study.

## RESULTS

The final analysis included 79 patients with a mean age of 59.8 ± 12.95 years. Most patients were male (63.3%), most patients had an infiltrated macula (89.9%), and most patients underwent PPV + SO injection (70.9%). Approximately one-third of the patients had a reported duration between symptom onset and surgery of fewer than 7 days (38.0%). Two-thirds of the patients had no prior history of previous intraocular surgery (63.3%), and nearly half of the patients were pseudophakic after RRD correction surgery (48.1%). The sample was considered homogeneous considering the incidence of ME; that is, there was no difference between the groups analyzed. Details are presented in table 1.

**Table 1:** Characterization of patients at baseline and differences in variables of interest based on the development of macular edema over 1 year of follow-up

Variables	Total sample (n=79)	Macular edema		p
		No (n=58)	Yes (n=21)	
Age, mean (SD)	59.8 (12.95)	58.2 (12.65)	64.3 (12.97)	0.060
Sex, absolute frequency (%)				
Male	50 (63.3)	37 (63.8)	13 (61.9)	0.539
Female	29 (36.7)	21 (36.2)	8 (38.1)	
Macular status, absolute frequency (%)				
Detached	71 (89.9)	51 (87.9)	20 (95.2)	0.674^*^
Attached	8 (10.1)	7 (12.1)	1 (4.8)	
PVR, absolute frequency (%)				
Yes	31 (39.2)	21 (36.2)	10 (47.6)	0.598
No	29 (36.7)	23 (39.7)	6 (28.6)	
Not determined	19 (24.1)	14 (24.1)	5 (23.8)	
Duration between symptom onset and surgery, absolute frequency (%)				
<7 days	30 (38.0)	23 (39.7)	7 (33.3)	0.760^*^
7 days-1 month	25 (31.6)	18 (31.0)	7 (33.3)	
1-3 months	17 (21.5)	11 (19.0)	6 (28.6)	
>3 months	7 (8.9)	6 (10.3)	1 (4.8)	
Surgery, absolute frequency (%)				
PPV + SO	61 (77.2)	44 (75.9)	17 (81.0)	0.767^*^
PPV + SO + PHACO + IOL	18 (22.8)	14 (24.1)	4 (19.0)	
Previous surgery, absolute frequency (%)				
Yes	29 (36.7)	19 (32.8)	10 (47.6)	0.226
No	50 (63.3)	39 (67.2)	11 (52.4)	
Lens status, absolute frequency (%)				
Phakic	37 (46.8)	28 (48.3)	9 (42.9)	0.549^*^
Pseudophakic	38 (48.1)	28 (48.3)	10 (47.6)	
Aphakic	4 (5.1)	2 (3.4)	2 (9.5)	

SD= standard deviation; PPV= pars plana vitrectomy; SO= silicone oil; PHACO= phacoemulsification; IOL= intraocular lens.

* Fisher’s exact test.

**Figure 1:**
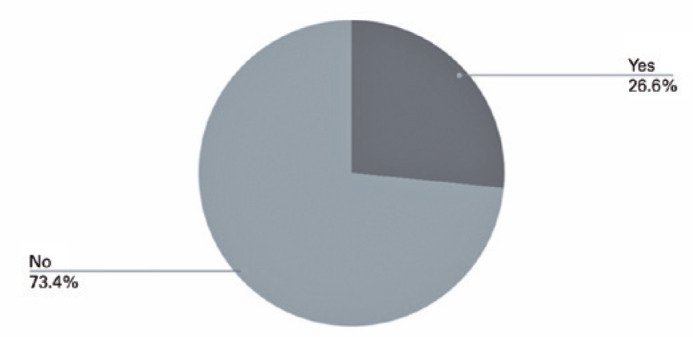
Prevalence of macular edema in a sample of 79 patients.

**Figure 2:**
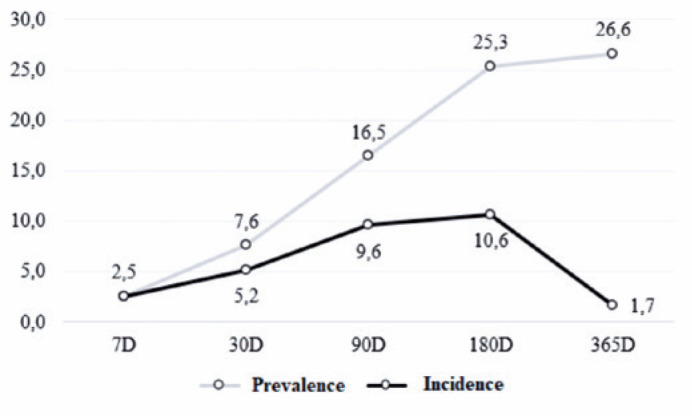
Prevalence and incidence rates of macular edema among patients over time.

The prevalence of ME at the end of the follow-up was 26.6% (21/79 patients), as presented in figure 1. Furthermore, the incidence of ME increased over time up to 180 days postoperatively, peaking at 10.6%, and then decreasing until 365 days postoperatively (Figure 2).

Regarding factors associated with the incidence of ME, logistic regression analysis identified no variable associated with outcomes. However, in the adjusted analysis, the model with the best fit (Hosmer-Lemeshow chi-squared = 0.519), which included six variables (age, sex, macular status, PVR, previous surgery, and intraocular lens status), demonstrated that age was associated with the incidence of ME. In addition, the analysis revealed that each 1-year increase in age increased the risk of ME by 6% (95% confidence interval = 1.00-1.12) regardless of the other variables included in the final model. Thus, age was considered a risk factor for the development of ME. No other independent variable was associated with ME. The results are presented in table 2.

**Table 2:** Factors associated with the incidence of macular edema after 1 year of follow-up

Variables	Macular edema		Raw OR (95% CI)	Adjusted OR^**^ (95% CI)
	No n (%)	Yes n (%)		
Age	58.2 (12.65)	64.3 (12.97)	1.05 (0.99-0.11)	**1.06 (1.00-1.12)**
Sex				
Male	37 (63.8)	13 (61.9)	0.92 (0.33-2.59)	1.2 (0.40-4.04)
Female	21 (36.2)	8 (28.1)	1	1
Macular status				
Applied	51 (87.9)	20 (95.2)	2.75 (0.32-23.76)	2.98(0.28-31.28)
Detached	7 (12.1)	1 (4.8)	1	1
PVR				
Yes	21 (36.2)	10 (47.6)	1.83 (0.57-5.90)	1.53(0.39-6.04)
No	23 (39.7)	6 (28.6)	1	1
Surgery performed				
PPV + SO	44 (67.7)	17 (81.0)	1.35 (0.39-4.69)	
PPV + SO + PHACO + IOL	14 (21.5)	4 (19.0)	1	
Previous surgery				
Yes	19 (32.8)	10 (47.6)	1.87 (0.68-5.16)	2.60(0.54-12.40)
No	39 (67.2)	11 (52.4)	1	1
Lens status				
Phakic	28 (48.3)	9 (42.9)	0.32 (0.04-2.62)	0.59(0.05-7.53)
Pseudophakic	28 (48.3)	10 (47.6)	0.36 (0.04-2.88)	0.24 (0.02-2.61)
Aphakic	2 (3.4)	2 (9.5)	1	1

Data are presented as mean (standard deviation).

OR = odds ratio; CI = confidence interval.

** The analysis was adjusted according to the best-fitting model.

When analyzing age groups, four categories were created according to the best delimitation of the sample (<50 years, 50-59 years, 60-69 years, and ≥70 years). Via regression analysis, age ≥70 years was identified as a risk factor for the development of ME (p=0.004); however, the confidence interval for this association was (extremely large (95% confidence interval = 2.09- -54.05). Possibly, this result was attributable to the small sample size. Thus, an age of 70 years or older istended to be associated with a higher risk of ME.

## DISCUSSION

The incidence of ME surgical correction of RRD has been reported as 8%-50% in the literature^(13)^. In patients with RRD who underwent PPV with SO injection, the incidence of ME ranged 19.6%-36.2%^(4^-^6)^. Our findings accorded with these results.

Although some studies did not identify significant asso-ciations of different risk factors with ME, others reported associations of ME with pseudophakic/phakic eyes, older age, the presence of an infiltrated macula and PVR findings, and the presence of RRD with more than 1 week between symptom onset and treatment^(8^,^13^-^16)^. In the present study, we found a higher incidence of ME in men, patients with an infiltrated macula, those with PVR, and patients with pseudophakia. However, these associations were not significant, and thus, these factors were considered possible risk factors for ME.

However, in the adjusted analysis, age was associated with a higher incidence of ME, and the risk of ME increased with increasing age. This finding is in line with data reported by Star et al., who analyzed 1466 eyes and identified an association of advanced age with the development of ME after PPV in patients with pseudophakia^(17)^. Meanwhile, Meredith et al. used fluorescein angiography to identify ME after RRD repair with the scleral buckle technique, and similarly as our study, they reported that older patients with phakia were more likely to develop ME^(16)^. In addition, Lai et al. analyzed 130 eyes submitted to RRD correction with the scleral buckle technique, and age was associated with the ME outcome in patients with phakia^(18)^. Age-related changes in retinal vessels can leave older patients vulnerable to manipulations during surgery, as well as alter vessel wall permeability, leading to a higher incidence of postoperative ME.

Prospective studies with more patients analyzed are necessary to confirm these results and better understand this pathology and its risk factors.

This study observed a significant prevalence of ME after surgical correction of RRD using PPV with SO infusion, with the incidence peaking between 30 and 180 days after surgery. Age was an important risk factor for ME in this cohort.
